# Versatile and non-versatile occupational back-support exoskeletons: A comparison in laboratory and field studies

**DOI:** 10.1017/wtc.2021.9

**Published:** 2021-09-21

**Authors:** Tommaso Poliero, Matteo Sposito, Stefano Toxiri, Christian Di Natali, Matteo Iurato, Vittorio Sanguineti, Darwin G. Caldwell, Jesús Ortiz

**Affiliations:** 1 Department of Advanced Robotics, Istituto Italiano di Tecnologia, Genova, Italy; 2 Dipartimento di Elettronica, Informazione e Bioingegneria (DEIB), Politecnico di Milano, Milan, Italy; 3 Department of Informatics, Bioengineering, Robotics and Systems Engineering, University of Genoa, Genova, Italy

**Keywords:** carrying, human activity recognition, lifting, manual material handling, occupational exoskeletons

## Abstract

Assistive strategies for occupational back-support exoskeletons have focused, mostly, on lifting tasks. However, in occupational scenarios, it is important to account not only for lifting but also for other activities. This can be done exploiting human activity recognition algorithms that can identify which task the user is performing and trigger the appropriate assistive strategy. We refer to this ability as exoskeleton versatility. To evaluate versatility, we propose to focus both on the ability of the device to reduce muscle activation (efficacy) and on its interaction with the user (dynamic fit). To this end, we performed an experimental study involving 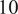 healthy subjects replicating the working activities of a manufacturing plant. To compare versatile and non-versatile exoskeletons, our device, XoTrunk, was controlled with two different strategies. Correspondingly, we collected muscle activity, kinematic variables and users’ subjective feedbacks. Also, we evaluated the task recognition performance of the device. The results show that XoTrunk is capable of reducing muscle activation by up to 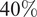 in lifting and 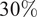 in carrying. However, the non-versatile control strategy hindered the users’ natural gait (e.g., 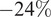 reduction of hip flexion), which could potentially lower the exoskeleton acceptance. Detecting carrying activities and adapting the control strategy, resulted in a more natural gait (e.g., 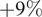 increase of hip flexion). The classifier analyzed in this work, showed promising performance (online accuracy > 91%). Finally, we conducted 9 hours of field testing, involving four users. Initial subjective feedbacks on the exoskeleton versatility, are presented at the end of this work.

## Introduction

**Figure 1.**
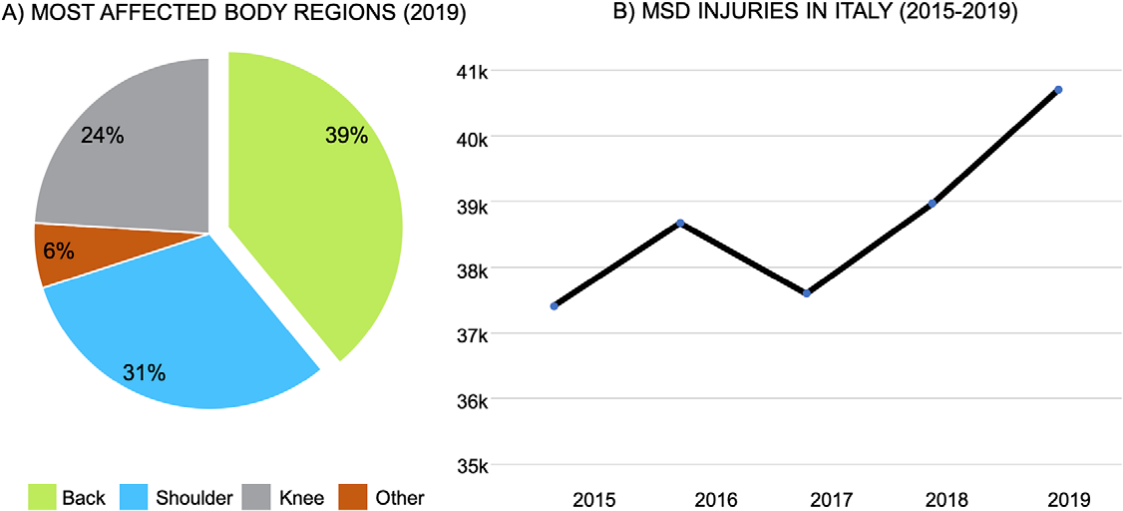
(a) The pie chart describes how MSDs have affected specific body regions in Italy, 2019. (b) The chart shows the number of occupational disease linked with MSDs, that occurred in Italy in the years 2015–2019.

Musculoskeletal disorders (MSDs) are physical diseases caused by over-exertion of the muscles at specific joints. The body region that is mostly affected by these disorders is the back, as shown in Figure 1a. MSDs can be triggered by several phenomena such as incongruous postures, handling of heavy loads and repetitive lifting (de Kok et al., 2019). Therefore, it is no surprise that workers performing *manual material handling* (MMH) activities (e.g., luggage handling in airports or sleepers replacement in railway construction) are among the most exposed to risks of injuries. Indeed, there are several biomechanical models that describe how spinal muscles and passive tissues generate, at the *L5-S1* joint, an extensor moment that grows proportionally with the trunk inclination and the weight of the payload (Chaffin, 1969; Toxiri et al., 2015; Koopman, 2020). Moreover, the greater the muscle activation, the higher the compression forces exerted on the lumbar disks (Dolan and Adams, 1993; Granata and Marras, 1995; Davis et al., 1998). If this compression exceeds specific biomechanical limits, the risk of developing MSDs increases (Moore and Garg, 1995). In the framework of MMH activities, these limits are easily exceed and, therefore, standards—like the ISO 11228—or technical solutions—like plant automation—have been introduced to mitigate the risk of MSDs. Unfortunately, the number of MSDs has not dropped, yet^1^ (see Figure 1b, for an example of the Italian trend). This might be caused not only by the high cost of some solutions but also by the users’ lack of adoption of new tools.

### Occupational Back-Support Exoskeletons

In the framework of MMH, many practitioners (e.g., ergonomists, engineers, H&S managers, and compensation authorities) have started evaluating *back-support exoskeletons* as a possible novel solution for reducing MSDs. More in particular, a 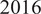 review on occupational exoskeletons reported that their usage was associated with up to 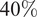 reduction in the activation of the back muscles, either considering repetitive lifting or static holding tasks (de Looze et al., 2016). As presented in section “Introduction,” the primary consequence of such reduction is a lower compression of the lumbar spine and, hence, a lower risk of injury. Such results are also confirmed by more recent works (Baltrusch et al., 2020; Heo et al., 2020; Kermavnar et al., 2020; Lanotte et al., 2021), but as warned in Theurel and Desbrosses (2019), an unreserved adoption of this technology is not justified yet, as the long-term effects on the working performance are still unclear. In this regard, in Lazzaroni et al. (2018) and Grazi et al. (2019), the authors present an overview of the methodologies used in the literature to evaluate the potential of back-support exoskeletons. Indeed, the authors identify that to compare different prototypes and foster promotion, it would be important to define a common evaluation method among the many proposed. Moreover, these works show that, in general, back-support exoskeleton evaluation focuses only on lifting activities, neglecting other relevant ones like carrying, pushing or pulling. However, the *International Standard ISO 11228* establishes ergonomic guidelines for all these activities, since they are considered to be associated with the MSDs onset. As an example, the NIOSH (National Institute for Occupational Safety and Health) engineering controls database^2^ reports that considering the compensable cases of low back pain associated with MMH tasks, *lifting* has been implicated in 37–49% of the cases; *pushing*, 9–16%; *pulling*, 6–9%; and *carrying*, 5–8%. In this regard, the work presented in Baltrusch et al. (2019), assesses the effects of a passive exoskeleton used not only in lifting tasks but also in walking. The results show that while the exoskeleton proved to be beneficial in the former tasks, it hindered the latter. This is due to the limitations of passive exoskeletons, which rely on purely mechanical elements—like gas/coil springs, flexible beams or elastic bands (Abdoli-e et al., 2006; Näf et al., 2018; Alemi et al., 2019). It follows that the exoskeleton user has to spend part of his/her energy, which is stored in the device and, then, released in successive phases. For such reason, passive devices try to find a trade-off between the energy absorbed and the provided assistance, by optimizing the output force/torque as function of a given movement. Therefore, the assistive performance drop as soon as activities, differing from the optimized one, are performed. On the contrary, active exoskeletons exploit controllable actuation elements—like electrical motors or pneumatic actuators (Aida et al., 2009; Toxiri et al., 2015; Yu et al., 2015; Inose et al., 2017). These actuators, rather than relying on the energy coming from the user, are powered by external sources (batteries or air tanks). Additionally, the combination of controllable actuators and on-board computers, allows to design specific assistive strategies according to the activity being performed. Therefore, it is necessary to find a mean to distinguish which activity the user is performing. In the literature, there are different examples of exoskeleton controllers that exploit human activity recognition (HAR) algorithms for this purpose (Kawai et al., 2004; Cevzar et al., 2018; Chen et al., 2018, 2019; Poliero et al., 2019; Jamšek et al., 2020). An exoskeleton that recognizes which task the user is performing and consequently triggers the appropriate control strategy is defined as *versatile.*

### Contributions of This Study

In this work, we show how versatile occupational back-support exoskeletons have an advantage with respect to nonversatile ones. This is done considering not only the device *efficacy*, but also its *dynamic fit.* Efficacy is studied as the ability to reduce muscle activation under controlled laboratory conditions (Singal et al., 2014), whereas addressing how the exoskeleton and its wearer move and interact with each other is what *dynamic fit* analyses (Stirling et al., 2020). Dynamic fit could strongly influence the workers’ adoption of a particular exoskeleton. Indeed, considering only efficacy might not properly capture the workers’ acceptance of the device. As an example, a passive exoskeleton with a very strong spring would drastically reduce muscle activation during lifting, but the users would struggle to move and interact with the exoskeleton. Therefore, such device could, theoretically, represent a very good tool for the reduction of MSDs but, most likely, it will never be used *out-of-the-laboratory.*

To show that versatile exoskeletons have better performance with respect to non-versatile ones, we devised an experiment divided in two phases: a laboratory study and a field test. In the laboratory, we replicated the typical working routine of workers in a food manufacturing plant. Access to this plant was possible thanks to a collaboration within the framework of the National project *Sistemi Cibernetici Collaborativi.* ^3^ We studied muscle activation, kinematic changes and subjective feedbacks collected from the 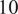 volunteers that took part in the experiment. To compare versatile and non-versatile exoskeletons, we controlled our back-support exoskeleton *XoTrunk* in two different ways: *XoLift* and *XoHar*, as presented below in section “Material and Methods.” As the name might suggest, this latter control strategy takes advantage of a HAR algorithm to switch between different control strategies. Instead, the former strategy uses a single control strategy. To the author’s knowledge, this is the first study in which an active back-support exoskeleton was used to assist a complex task, including both lifting and carrying activities. section “Results and Discussion” describes the obtained results for each of the metrics presented in section “Evaluation Metrics.” For the sake of simplicity, the presentation of each single results is followed by its discussion. Section “Field Testing” describes the preliminary feedback collected on the versatile exoskeleton performance during field testing. In particular, we had the opportunity to involve four workers for a duration of 9 hours, while performing the task simulated in the laboratory. Finally, section “Conclusion” concludes this work.

## Material and Methods

The experiment performed in this study, approved by the Ethics Committee of Liguria, Italy (protocol reference number: CER Liguria 001/2019), was conducted on 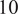 subjects (five males, five females, 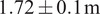 height, 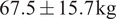 weight, 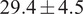 years old, and no previous history of back pain).^4^ None of the experimental subjects had prior experience in back-support exoskeletons usage. The choice of inexperienced volunteers was done to collect impressions that could provide a first indication on how the workers would react to the usage of a new device.

### Task Description

The subjects were required to perform a task that simulates an actual working activity. In particular, in the scenario under analysis, the workers perform not only lifting, but also carrying and unloading of animal parts. In the plant, the weight of the payloads varies in a range between 6 and 15 kg. Additional complexity is related to the animal parts being slippery and not easy to handle. In the laboratory replica, the subjects were instructed to perform the following sequence of actions:

1. starting from a standing position, *reach* for a payload (W, 11.4 kg) placed on a surface (B1) at 0.5 m height from the ground;
2. *lift* the payload with self-chosen motion style and return to an upright posture;
3. *turn* 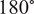 to change direction;
4. *carry* the weight for a 3.5 m distance until a second surface (B2) is reached;
5. *lower* the weight onto B2, at 0.5 m height from the ground;
6. *return* to upright posture;
7. rest in *standing* position.

**Figure 2.**
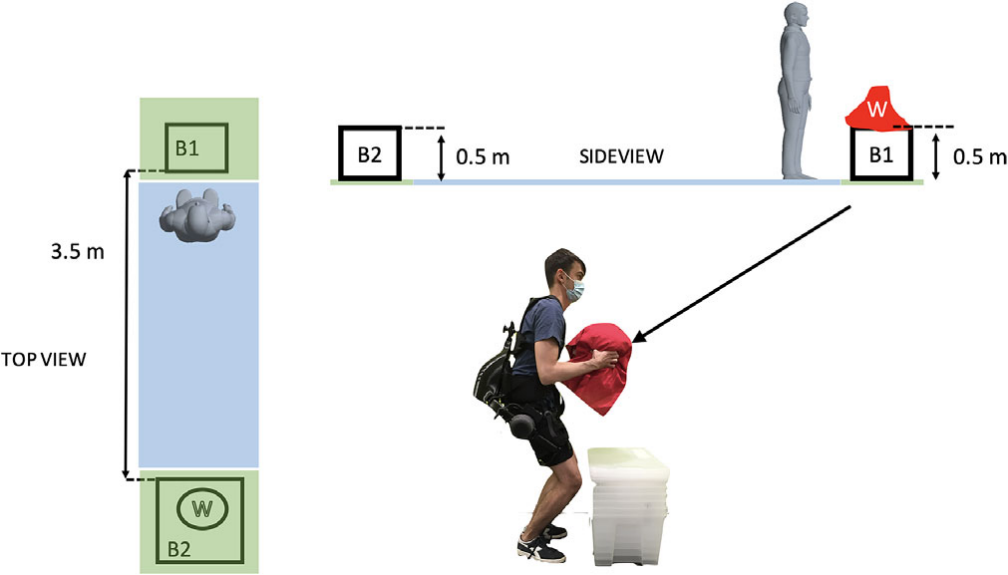
Schematic representation of the experimental task. W represents the 11.4 kg weight, B1 and B2 are the two boxes onto which the weight was placed. The carrying task was performed within the light blue area (3.5 m) and the lifting one in the light green zones. Note that to move from B1 to B2 and vice versa, the subject needs to perform a 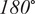 rotation. At the bottom, the picture shows a subject while holding the load and wearing XoTrunk, the active back-support exoskeleton used in this assessment.

Sequence 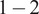 is referred to, in the following, as *lifting*, 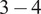 as *carrying*, 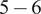 as *lowering*, and 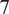 as *standing.* When switching from an action to the following, the subjects were asked to verbally express their intention. This information was then used for the labeling of the activity. Furthermore, the subjects were given 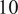 s to complete the whole sequence. The frequency matched that of the actual working operation. The lifted object was a backpack stuffed with foam and payloads, and covered with fabric. This was done in order not to offer evident handling points and, again, to match the industrial scenario. Figure 2 summarizes the task description. The task was repeated 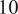 times for each of the three following conditions:

- *noExo*: subject not wearing the exoskeleton;
- *XoLift*: subject wearing XoTrunk exoskeleton constantly providing assistance;
- *XoHar*: subject wearing XoTrunk exoskeleton switching between assistance and transparency, based on HAR;

Section “XoTrunk: An Active Back-Support Exoskeleton” reports more details about the exoskeleton and the control conditions. No rest phase was planned between each of the 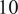 task repetitions, so that the faster the *lifting–carrying–lowering* sequence execution, the longer the standing phase duration. Following the 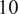 s interval, the subjects were instructed to start with the successive repetition. Fifteen minutes of rest were planned between the three conditions. During the resting time, the subjects filled-in a custom questionnaire, as presented in section “Data Processing.” Even though the task sequence, the execution frequency and the payload form-factor were selected in order to mimic the industrial scenario, changes were done in the heights and the movement constraints to ensure safety of the test volunteers. Also, the 11.4 kg weight of the payload was selected, in a [6–15] kg range, in order to ensure safety. In particular, the NIOSH lifting index associated with the simulated activity was 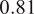 for males, and 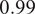 for females (Waters et al., 1993). Since both these values are less than 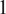 , the task can be considered safe.

### XoTrunk: An Active Back-Support Exoskeleton

**Figure 3.**
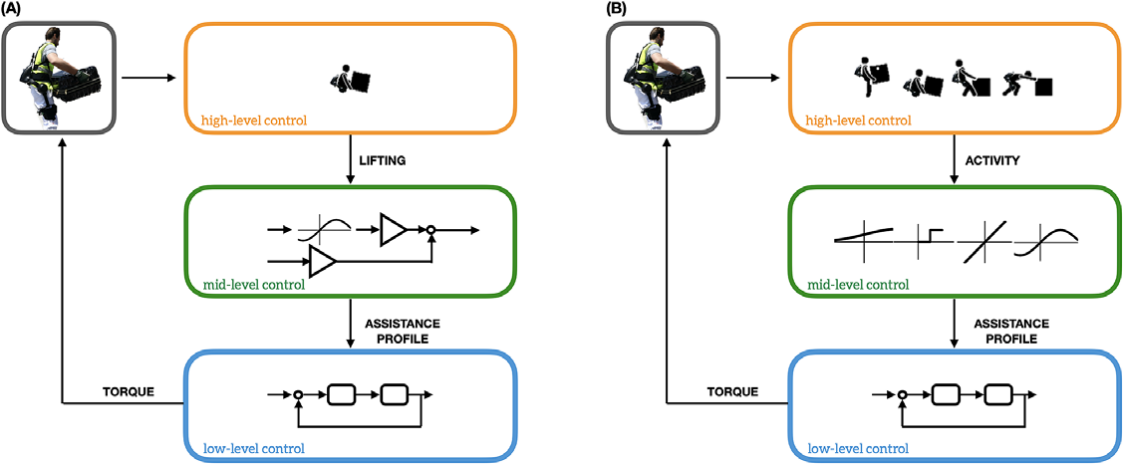
Schematic representation of (a) a nonversatile controller that assumes the user is always performing lifting and (b) a versatile one that detects additional relevant MMH activities like carrying, pushing, and pulling. Note how in (a) the mid-level control (green box) only implements one control strategy, whereas in (b) multiple control strategies are designed to assist the different activities.

*XoTrunk* (see Figure 2) has an overall 6.5 kg weight (including batteries) and it is worn like a trekking backpack with additional attachments on the user’s thighs. The rigid frame houses the anchoring point for the actuation units (one per side, at hip level, each including a brushless DC motor and reduction gear), the electronics and the lumbar cushion for improving comfort and dissipating the exoskeleton reaction force. The actuators are torque-controlled and, when commanded, they deliver assistance in the sagittal plane. Additional passive degrees of freedom allow unhindered movements outside the plane of interest. A more detailed analysis of how misalignments in the *XoTrunk* kinematic chain can negatively influence physical comfort and might compromise users’ acceptance, is presented in Sposito, Di Natali, et al. (2020). The control of *XoTrunk* can be divided into three levels, as defined in Tucker et al. (2015). The low-level control minimizes the desired torque ( 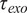 ) tracking error. More details about the implementation of this level, and about the actuators used to generate the torque can be found in Di Natali et al. (2020). The middle level generates appropriate values for the desired torque, relying on several inputs such as trunk inclination, trunk acceleration and myoelectric activity of the forearm (Toxiri et al., 2018; Lazzaroni et al., 2020). For the sake of simplicity, in the assessment studies presented hereafter, the trunk acceleration component is neglected from the torque reference generation. The torque profile is designed to be assisting with hips and trunk extensions and does not distinguish between right or left actuator, meaning that the delivered profile is symmetrical. The high-level, responsible for recognizing the activity that is performed by the user, is based on the algorithm that was presented in Poliero et al. (2019). *XoTrunk* was controlled according to two different strategies:

- *XoLift*: only middle and low level of control are used. Thus, regardless of the activity performed, assistance is always computed combining trunk inclination and forearm activity. This is the same control strategy that was used in Poliero et al. (2020) when investigating whether back-support exoskeletons could be used also to assist with carrying tasks. The XoLift control strategy is designed considering only lifting tasks, just like as if the high-level could only recognize this latter activity, as represented in Figure 3a.
- *XoHar*: all the three levels of control are used. More in details, if a *lifting* or *lowering* activity is recognized, the exoskeleton provides assistance modulating the output torques via the two lower levels of control, as in *XoLift.* However, if a *carrying* activity is recognized, the exoskeleton enters *transparency* mode. This strategy commands for torques that generate null torque interaction between user and exoskeleton, so that no assistance is provided. As discussed in Poliero et al. (2020), this strategy is expected not to hinder the natural gait. XoHar takes full advantage of the three-level control strategy, as represented in Figure 3b.

For both conditions, in lifting and lowering, the mid-level control strategy was set to:
(1)

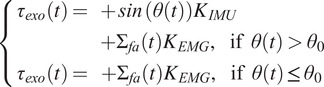

where 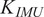 and 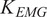 were equal to 13 Nm and 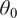 to 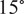 . This angle offset allows unassisted movements for 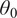 degrees if no load is handled. 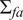 represents the forearm muscle activation and, as in a previous study (Toxiri et al., 2018), it equals 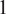 whenever the load is handled. On the other side, during carrying, XoLift provides an extensive torque 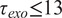 nm per side, whilst XoHar is commanded in transparency.

### Evaluation Metrics

First, we present metrics that can be used to assess the device efficacy and then those for evaluating the dynamic fit.

#### Efficacy

In this study, the 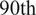 ( 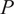 ) and the 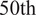 ( 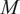 ) percentile of surface EMG signal distributions are taken into account. Such choice is justified by the work presented in Jonsson (1982). Here the authors show that 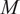 can be associated with how the muscle has been working during the activity, whilst 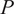 can be associated with a noise-less estimation of the maximum muscle activity. These two metrics are relevant as they can monitor the risk associated with cumulative fatigue and traumatic damages, respectively.

It is worthwhile pointing out that because of their versatility, back-support exoskeletons can have effects also on tasks differing from lifting. For such reason, in this work, efficacy is assessed considering not only lifting, but also carrying and lowering tasks. Splitting the bending motion analysis into lifting and lowering could additionally highlight that—exploiting the assistance modulation according to the task—active and versatile exoskeletons might have higher potential than nonversatile ones.

#### Dynamic fit

How the exoskeleton and its wearer move and interact with each other is defined as dynamic fit (Stirling et al., 2020). There are many different metrics that can be taken into account. Therefore, in this study, we focus on the final user’s capability to continue working in a natural way and receive appropriate assistance, according to the task. More in particular, the proposed metrics are:

- *Kinematics*: back-support exoskeletons deliver forces that act on the users’ hips and trunk. Therefore, to understand how the exoskeleton action is changing the users’ movements, it is possible to consider changes in the extension and flexion of the previous joints. In particular, for lifting and lowering, analyzing the maximum flexion ( 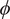 ) reached by the trunk and the hip joints could provide information on postural changes during the bending movement. Taking carrying into analysis, instead, it might be interesting to consider how the exoskeleton is affecting not only 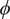 , but also the minimum extension ( 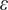 ) angle reached by the trunk and the hips. Indeed, this is expected to highlight advantages or drawbacks of the exoskeleton assistance during carrying.
- *Intuitiveness and ease of use*: the introduction of versatile capabilities in the exoskeleton should not affect how the workers normally perform their jobs. For instance, if the workers are required to manually switch between different control strategies, this could negatively impact on the use of a device in a MMH framework. On the other hand, relying on automatic switching could be worse than manual one, if it is not working properly. Those aspects can be assessed through questionnaires—relying on well-established tests like SUS or Nasa TLX or on custom defined ones (Shore et al., 2020; Sposito et al., 2020).
- *Activity recognition performance*: the first check should be on the device or the user’s capability to recognize the activities being performed. Secondly, the assessment should consider how long it takes for the device, or the user, to recognize that a given activity is being performed. Relevant metrics to answer these questions are represented by confusion matrices (CM) that identify precision, recall, and accuracy, and the estimation time delay (ETD).

As for the efficacy analysis, the above-mentioned metrics should be evaluated for each of the user activities.

### Data Collection

As proposed in section “Efficacy,” to assess efficacy, sEMG signals were recorded bilaterally at 1 KHz sampling frequency from both *Erector Spinae Longissimus* and *Iliocostalis* muscles. The acquisition was done by means of surface electrodes (BTS FREEEMG, BTS Bioengineering, Italy). The electrodes placement was based on SENIAM guidelines.^5^ Prior to the task execution start, maximum voluntary contraction (MVC) values for these muscles were acquired.

To assess kinematic metrics, motion data were collected thanks to a 3D motion tracking system (MTw Awinda, Xsens, The Netherlands), at a 60 Hz sampling frequency. To track lower limbs and trunk movements, eight inertial trackers were placed, respectively, on the sternum, the pelvis, right and left thighs, shanks and feet. Before the task start, the Xsens calibration routine was performed.

For the classification performance evaluation, during the task execution, the experimenter manually performed labeling of the activity carried out by the subject, classifying it as *lifting*, *carrying*, or *standing.* To facilitate the manual labeling, the subject was required to spell out each of the performed activities. From the classifier point of view, both lifting and lowering tasks were classified as *lifting.* This choice was related to the fact that the same strategy was used to assist with these activities (see section “XoTrunk: An Active Back-Support Exoskeleton”).

Acquisitions of sEMG signals, motion data, manual activity labeling and exoskeleton data were all synchronized, via TTL signals.

Additionally, even though manual labeling did not discriminate between lifting and lowering (as defined in section “Task Description”), this was possible taking advantage of how the task was performed. Indeed, each lifting always preceded a lowering. Therefore, all the even sequences labeled as lifting, were actually associated with lowering.

At the end of each tested condition (excluded the *noExo* one), the subjects were asked to fill-in a custom seven anchor Likert-scale questionnaire answering the following questions.

1. Overall, do you feel less fatigued with respect to the noExo (control) condition?
2. Did you feel that the exoskeleton action was constraining/hindering the natural leg movements, with respect to the noExo (control) condition?
3. Did you feel that the exoskeleton action was providing assistance during the carrying activity?
4. Did you feel that the exoskeleton action was constraining/hindering the natural trunk movements, with respect to the noExo (control) condition?
5. Did you feel that the exoskeleton action was providing assistance during the lifting activity?
6. Is it useful that the exoskeleton automatically recognizes the activity being performed?
7. How often would you like to manually set the activity being performed?

Questions 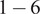 were associated with the following anchors: *Totally Disagree*, *Somewhat Disagree*, *Slightly Disagree*, *Neutral*, *Slightly Agree*, *Somewhat Agree*, *Totally Agree.* Question 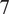 , instead, with the following ones: *Never*, *Rarely*, *Seldom*, *Sometimes*, *Occasionally*, *Frequently*, *Always.*

### Data Processing

The data processing is divided in two parts: efficacy and dynamic fit.

#### Efficacy

First, sEMG signals were band-pass filtered (35–350 Hz), smoothed and rectified in order to extract envelopes, and subsequently normalized with respect to MVC values. Second, the overall lumbar extensor activity was computed averaging the right and left side activity of the *Erector Spinae Longissimus and Iliocostalis* muscles, as in Koopman et al. (2019). Therefore, all the efficacy analysis was done with respect to this new variable. Third, the extensor activity was segmented, based on the manual activity labeling, into lifting and carrying. Since each subject performed 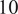 times the *lifting–carrying–lowering* sequence, at the end of data segmentation, 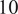 carrying segments and 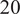 lifting segments were obtained, for each subject. These latter 20 segments were further divided in lifting and lowering, as described in section “Data Collection.” Fourth, to consider only steady state carrying, for this task the analysis was limited to data belonging to the [ 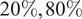 ] time-interval of each sequence. Fifth, 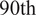 ( 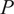 ) and 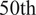 ( 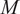 ) percentiles of the EMG signal distribution were calculated for each segmented data. Eventually, these metrics were grouped and averaged according to activity and condition (*noExo*, *XoLift*, and *XoHar*).

Similarly to what was presented in Lamers et al. (2020), we introduced an acceptance criterion for the muscle activation data. In particular, all data associated with exoskeleton conditions that, during lifting, did not result in P reductions in the 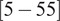 percentage range, were discarded. The idea behind this approach was that the interval defined above would capture EMG reductions related to the efficacy of the device and possible subject variability. Any data outside this interval, indeed, was assumed to be caused by issues such as sweating or electrodes displacement, rather than the exoskeleton action. The 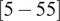 percentage range was defined considering that previous studies on the device (Huysamen et al., 2018; Toxiri et al., 2018; Lazzaroni et al., 2020) found its efficacy being around 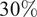 . To account for subject variability, we added a 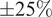 interval to the expected efficacy.

#### Dynamic fit

As seen before, dynamic fit includes several metrics that, for processing purposes, can be divided into three categories: kinematic data, exoskeleton data, and subjective data.

##### Kinematic data

The joints under analysis were hip (left and right) and trunk. The hip angles were defined as the relative orientation between the thighs and the pelvis, whereas the trunk angle was defined as the absolute orientation of the sternum. These joint trajectories were reconstructed by means of the Xsens system^6^ and segmented based on the manual activity labeling performed during the experiment. Again, data segmented as lifting were further discriminated into lifting and lowering. For each carrying segment, the analysis was limited to data belonging to the [ 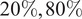 ] time-interval. Then, 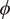 and 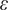 were computed for each individual phase and, successively, averaged according to activity and condition. Left and right hip angles were averaged together. Therefore, the following metrics were analyzed: 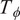 , 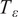 , 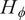 , and 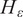 , considering Trunk (T) or averaged left and right Hip (H).

##### Exoskeleton data

To evaluate the activity recognition performance, the classification confusion matrix (CM) was calculated. The manual labeling was taken as the true class, whereas the activity recognized by the exoskeleton as the predicted one. Precision ( 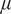 ), recall ( 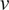 ), and accuracy ( 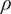 ) were then calculated. For each activity transition, the ETD was computed as the difference between the occurrence of a manually labeled transition and the corresponding automatic one. The values obtained for transitions of the same type were, then, averaged. If the automatic transition precedes the manual one, ETD is negative. However, to simplify the analysis, the absolute values of each transition was considered.

##### Subjective evaluation

Subjective data was collected by means of the questionnaire presented in section “Data Collection.”

### Statistical Analysis

In this experiment, the same participants are observed under three different conditions resulting in a within-subjects study design. For such reason, if data met the normally distributed condition (Lilliefors test; Ghasemi and Zahediasl, 2012), a one-way repeated ANOVA was conducted with control strategy (noExo, XoLift, XoHar) as within-subject factors. On the contrary, for those data that were not normally distributed, we applied the Friedman test. For metrics with a significant effect of the control strategy (*p*-value 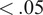 ), post hoc Bonferroni tests were conducted, to compare the effects of the different control strategies. This analysis was conducted on both the muscle activation metrics and the kinematic metrics. Since the questionnaire was based on Likert data that are ordinal, discrete, and have a limited range, a nonparametric Wilcoxon signed rank test was conducted (significance level set at 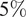 ).

Statistical analysis was performed with the Matlab 2019a (The Mathworks, Natick, MA) and R v3.3.3 (R Foundation for Statistical Computing, Vienna, Austria) software.

## Results and Discussion

Results are presented first for what concerns efficacy and, then, dynamic fit. Given the amount of metrics, for the sake of clarity, punctual discussions are reported right after the result of interest. Table 1 reports the *p*-values of the one-way repeated ANOVA with within-factor the control strategy (noExo, XoLift, XoHar). For each of the analyzed metrics, significant values are reported in *bold.* The apex 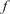 indicates those data for which a Friedman test was conducted. For lifting and lowering activities, the extension angle of the trunk ( 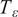 ) and the hips ( 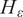 ) were not analyzed, as described in section “Dynamic fit.”

**Table 1.** *p*-values of the one-way repeated ANOVA with within-factor the control strategy (noExo, XoLift, and XoHar)

Activity	Metrics					
	*P*	M	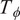	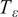	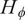	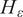
Lifting	**<.001**	**<0.05** 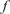	<0.01	n.a.	<0.15	n.a.
Carrying	**<0.001**	**<0.05** 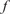	**<0.05** 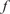	<0.25	**<0.001**	<0.1 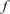
Lowering	<0.01	**<0.001**	<0.15	n.a.	<0.25	n.a.

*Note:* Significant values are in *bold.* The apex *f* indicates those data for which a Friedman test was conducted. For lifting and lowering activities, the trunk (*T_ε_*) and the hips (*H_ε_*) extension angles were not analyzed, as described in section “Dynamic fit.”

### Efficacy

**Figure 4.**
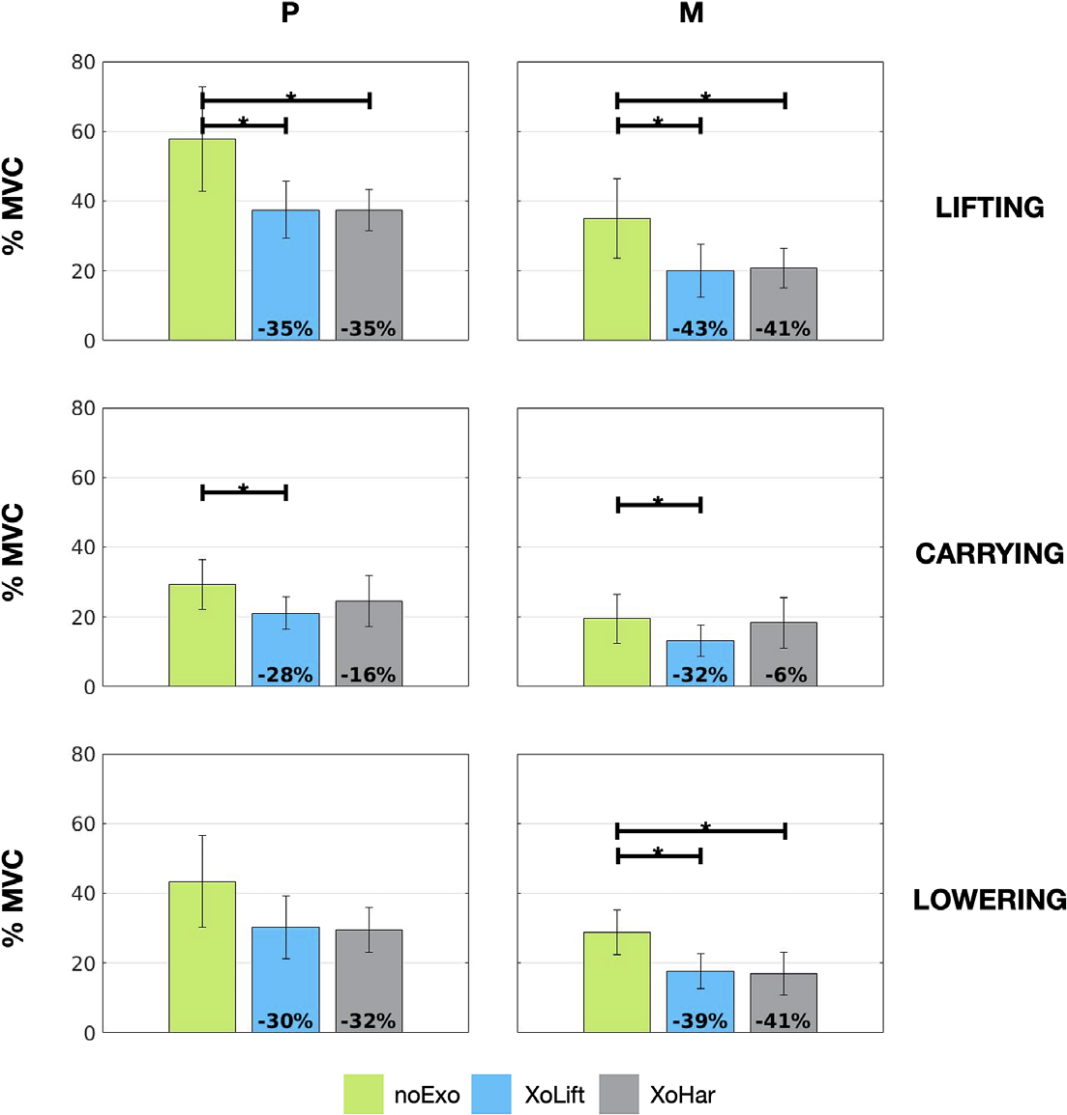
Exoskeleton efficacy on overall lumbar activity. The bar graphs represent the mean muscle activation considering P (left column) and M (right column). Vertical segments represent the variability given by the standard deviation, whereas horizontal segments connect those conditions for which the post hoc Bonferroni tests reported a statistically significant difference ( 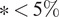 ). The noExo conditions are represented in green, whereas light blue and light gray were chosen to represent the XoLift and the XoHar conditions, respectively. In the conditions with the exoskeleton, the numbers at the bottom of the graph report the relative variation of the considered metric with respect to the noExo condition. Finally, each row of the figure represents one different activity. From top to bottom: lifting, carrying, and lowering.

Considering the EMG data, out of the initial pool of 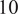 subjects, 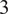 were excluded. The first because muscle reduction was lower than 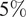 and the other two because their muscle reductions were around 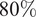 (see section “Data Processing”). Table 2 presents the obtained results (mean 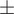 standard deviation) as percentage of the MVC of the overall lumbar extensor. The results are reported for each metric under analysis ( 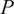 and 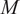 ), for each control condition (noExo, XoLift, and XoHar) and for each activity (lifting, carrying, and lowering). Figure 4 provides a graphical representation of the results. Also, the figure highlights by means of interconnecting segments, those conditions for which the post hoc Bonferroni tests reported a statistically significant difference.

**Table 2.** Mean ± standard deviation values of the overall lumbar extensor *90th* percentile (P) and *50th* percentile (M), expressed as a percentage of the MVC

Activity	P [ 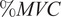 ]			M [ 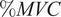 ]		
	noExo	XoLift	XoHar	noExo	XoLift	XoHar
Lifting	58 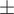 15	38 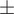 8	37 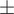 6	35 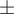 11	20 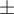 8	21 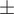 6
Carrying	29 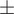 7	21 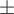 5	25 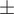 7	20 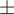 7	13 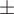 4	18 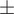 7
Lowering	43 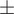 13	30 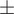 9	30 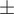 6	29 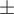 6	18 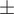 5	17 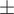 6

*Note:* Each row reports the values obtained during the execution of the three different activities (lifting, carrying, and lowering).

Analyzing the results, it is interesting to note that in noExo condition, considering the P metric, the activity that has the highest impact is lifting (mean value of 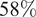 ), followed by lowering (mean value of 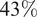 ), and carrying (mean value of 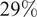 ). Similar findings are found focusing on the M metric. These results confirm the outcome of the study presented in Poliero et al. (2020), where carrying was found to have a relevant impact on spinal loading, compared to lifting. Also, investigating the standard deviation values, it is interesting to point out how the noExo condition is more variable than XoLift and XoHar ones. This might indicate that XoTrunk assistance well adapts to different subjects.

Analyzing Table 2 and focusing on the lifting and lowering outcomes, these confirm that XoTrunk is capable of remarkable overall lumbar activity reductions (up to 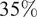 relative reduction considering P and 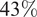 relative reduction considering M), as presented in previous studies. Given that for both conditions (XoLift and XoHar) the mid-level control strategy was the same, it is not surprising that the results are almost equivalent. However, there are some subtle differences (up to 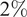 ). These could be explained by changes in postures and delays of the classifier in providing assistance. The statistical analysis shows that in lifting and lowering (only considering M), both XoLift and XoHar conditions were statistically different from the noExo one. Moreover, no significant differences between the two conditions with the exoskeleton were found, as expected.

On the other hand, considering carrying, it is interesting to point out that only the XoLift control strategy produces relative variations, with respect to noExo, that reach statistical significance. This result strengthen the analysis of the trends discussed in our previous study (Poliero et al., 2020), proving that back-support exoskeletons can be used to assist also with carrying activities. In particular, analyzing the XoLift condition, the results report a 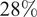 relative decrease in the P metric and a 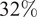 relative reduction in the M one, in line with the reduction levels found in lifting and lowering. Instead, in the XoHar condition, considering the P metric, the muscle reduction levels are lower than the noExo case ( 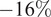 relative variation), while they are less consistent considering the M metric ( 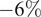 relative variation). This is due to the change in control strategy that, in the XoHar case, commanded for null interaction torques, in order not to hinder the users’ movements. This choice apparently penalizes the device efficacy, but improves its dynamic fit (as discussed in the following sections). A final remark is on the 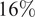 relative reduction of P obtained during carrying in the XoHar condition. This could be linked with postural or speed changes, also suggesting that the backwards push of the exoskeleton during lifting, might help maintaining better carrying postures.

### Dynamic Fit

First kinematic data, second classifier performance and third results associated with the subjective questionnaire are presented.

#### Kinematic data

**Figure 5.**
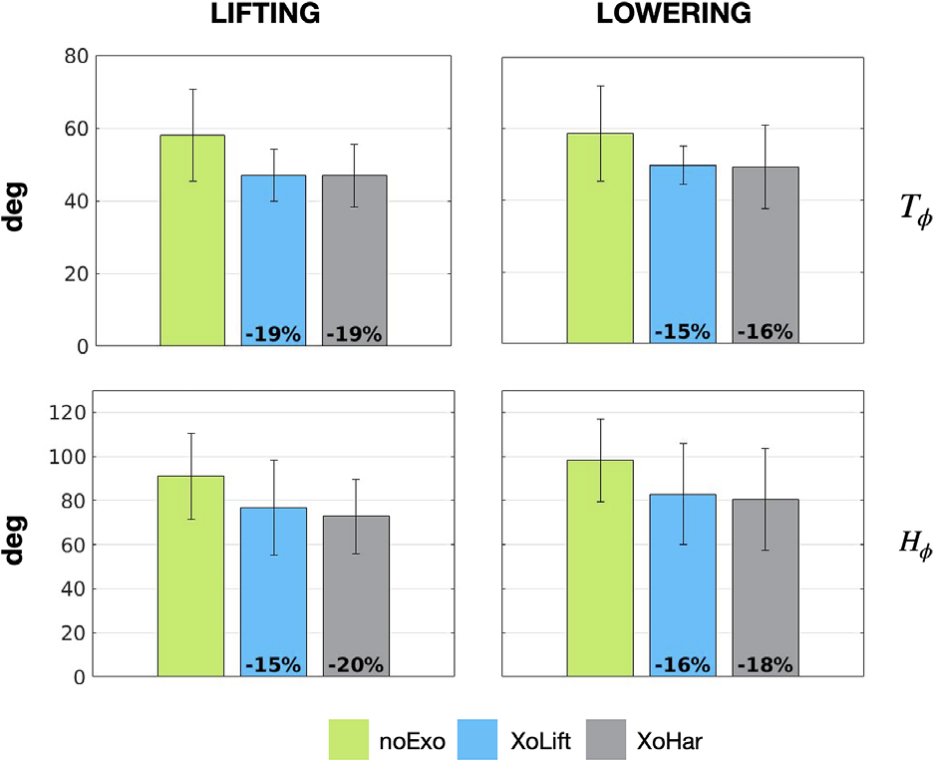
Trunk and hip kinematics during lifting and lowering. The bar graphs represent the maximum flexion ( 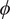 ) reached by the trunk (top row) and the hips (bottom row), for lifting activities (left column), and lowering activities (right column). Vertical segments represent the variability given by the standard deviation. The noExo conditions are represented in green, whereas light blue and light gray were chosen to represent the XoLift and the XoHar conditions, respectively. In the conditions with the exoskeleton, the numbers at the bottom of the graph report the relative variation of the considered metric with respect to the noExo condition.

**Figure 6.**
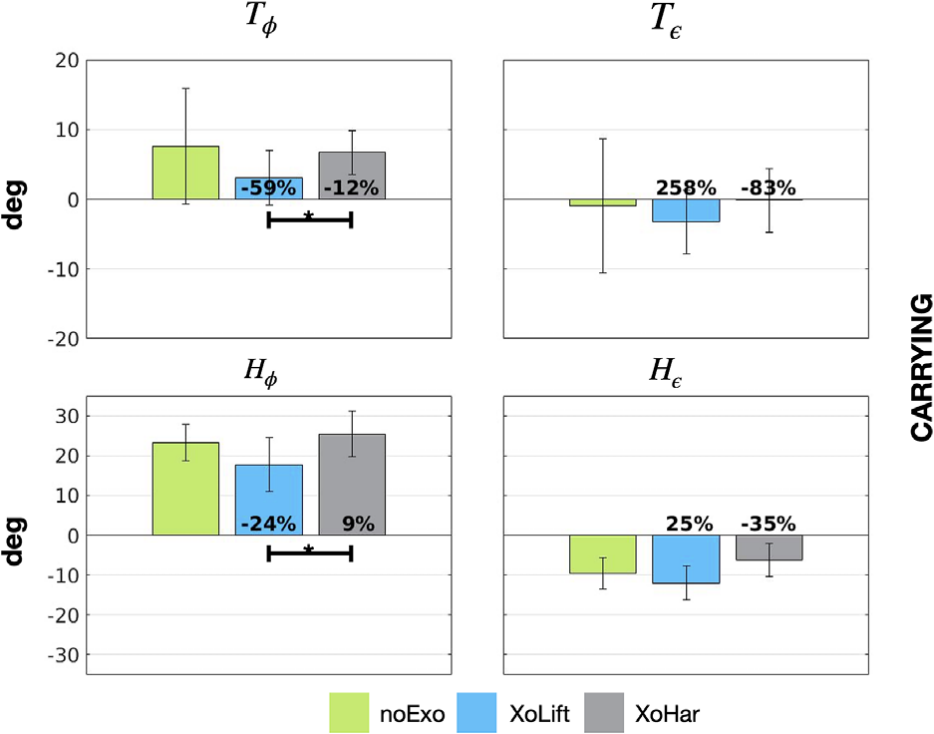
Trunk and hip kinematics during carrying. The bar graphs represent the maximum flexion ( 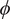 ) and extension ( 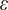 ) reached by the trunk (top row) and the hips (bottom row), during carrying. Vertical segments represent the variability given by the standard deviation, whereas horizontal segments connect those conditions for which the post hoc Bonferroni tests reported a statistically significant difference (* < 5%). The noExo conditions are represented in green, whereas light blue and light gray were chosen to represent the XoLift and the XoHar conditions, respectively. In the conditions with the exoskeleton, the numbers at the bottom of the graph report the relative variation of the considered metric with respect to the noExo condition.

Tables 3 and 4 summarize how the flexions and the extensions of trunk and hip joints varied across tasks and conditions, respectively. Moreover, Figures 5 and 6 depict (similarly to what was done in Figure 4), the obtained results.

**Table 3.** Mean ± standard deviation values of the trunk flexion (T_ϕ_) and extension (T_ε_), expressed in degrees

Activity	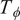 [deg]			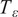 [deg]		
	noExo	XoLift	XoHar	noExo	XoLift	XoHar
Lifting	58 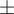 13	47 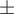 7	47 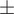 9	n.a.	n.a.	n.a.
Carrying	8 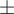 8	3 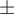 4	7 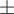 3	−1 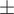 10	−3 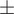 5	0 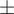 5
Lowering	59 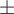 13	50 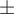 5	49 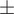 12	n.a.	n.a.	n.a.

*Note:* Each row reports the values obtained during the execution of the three different activities (lifting, carrying, and lowering). For lifting and lowering, the *T_ε_* metric was not analyzed, as reported in section “Dynamic fit.”

**Table 4.** Mean ± standard deviation values of the hip flexion (H_ϕ_) and extension (H_ε_), expressed in degrees

Activity	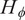 [deg]			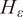 [deg]		
	noExo	XoLift	XoHar	noExo	XoLift	XoHar
Lifting	91 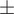 20	77 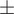 22	73 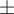 17	n.a.	n.a.	n.a.
Carrying	23 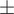 5	18 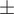 7	25 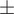 6	−10 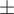 4	−12 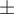 4	−6 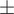 4
Lowering	98 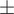 19	83 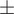 23	81 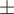 23	n.a.	n.a.	n.a.

*Note:* Each row reports the values obtained during the execution of the three different activities (lifting, carrying, and lowering). For lifting and lowering, the *H_ε_* metric was not analyzed, as reported in section “Dynamic fit.”

Up to this point, taking only in consideration the device efficacy, the advantage of a versatile exoskeletons does not emerge. On the other hand, this changes once dynamic fit is analyzed. In particular, focusing on the trunk, the results show that the exoskeleton usage (both XoLift and XoHar) reduced the trunk flexion by up to 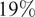 , both for lifting and lowering (see Figure 5). The differences between noExo and XoLift/XoHar did not reach statistical significance, but show a reductive trend. At first, the decrease in the flexion of the trunk could be interpreted as having a negative impact on the user. However, a deeper analysis changes the perspective. Indeed, a reduced trunk flexion yields more ergonomic postures, reduces the risk of flexion relaxation (Koopman et al., 2019) and could indicate that, thanks to the assistance provided by the device, the users felt less need of bending. Also, these postural changes can be considered one of the factors that determined reduction of the back muscle activation, during lifting and lowering. Considering lifting and lowering, also the analysis of the hips kinematics aligns with the previous results. In particular, even though the data was found not to be statistically significant, there is a clear reductive trend (up to 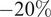 ) for both XoLift and XoHar. This might suggest that the subjects had to perform shallower squatting movements, and this can be associated with a reduced metabolic consumption (Hagen et al., 1993). The most evident differences between the versatile and the non-versatile controller are found analyzing carrying. Figure 6 stresses that the XoLift strategy, taking advantage of the backwards push of the device, could support a more ergonomic posture of the trunk, almost zeroing the trunk flexion ( 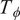 ). This was not possible in the versatile condition and the differences between the two control strategies were statistically significant. However, even though the XoLift backwards push assisted with trunk flexion ( 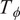 ), it also caused an hyperextension, that, on the contrary, is zeroed by the versatile controller. Here, the data does not reach statistical significance and, as shown in Figure 6, the variability is notable. Nevertheless, it is possible to argue that a control strategy that causes hyperextension could produce lower acceptance by the user.

The hypothesis made in Poliero et al. (2020) was that a constant assistance as that provided by XoLift would be beneficial for the back (as supported by efficacy results) but hinder the natural gait. On the contrary, switching to transparency mode, would diminish such obstacle. This is clear by analyzing the hip flexion angles ( 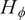 , 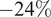 reduction in XoLift and 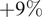 increase for XoHar). Indeed, even though the conditions with the exoskeleton were not statistically different from the noExo one, possibly due to the data variability, the previous results highlight how XoHar is closer to the noExo values than the XoLift condition. In particular, this latter control strategy, due to the constant backward push of the device, reduces the hip flexion, whereas commanding for null interaction torques, the XoHar controller does not hinder the motion. Also, differences between XoLift and XoHar were found to be statistically significant. Additionally, the hip extension angles show a trend for which the XoLift control strategy might cause an increase in the extension angle, caused by the exoskeleton action. On the contrary, the XoHar condition presents a slight reduction of the extension angle, possibly indicating that the exoskeleton, even if transparent (measured residual torques 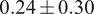 nm), might have partially hindered the gait. In conclusion, while the efficacy analysis showed the advantage of providing constant assistance during the carrying task, the kinematic analysis shows that the versatile controller is more respectful of the natural gait patterns, possibly resulting in an increased acceptance by the workers.

#### Classifier performance

Analyzing the Confusion Matrix (CM) reported in Table 5, it is possible to extract the classifier performance.

**Table 5.** Classifier online performance

	Standing	Lifting	Carrying	Precision ( 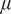 ) (%)
Standing	3,305	174	9	94.75
Lifting	274	3,539	257	86.95
Carrying	2	164	2,911	94.61
Recall ( 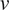 ) (%)	92.29	91.28	91.63	

*Note:* Rows represent the true output and columns the predicted output.

**Figure 7.**
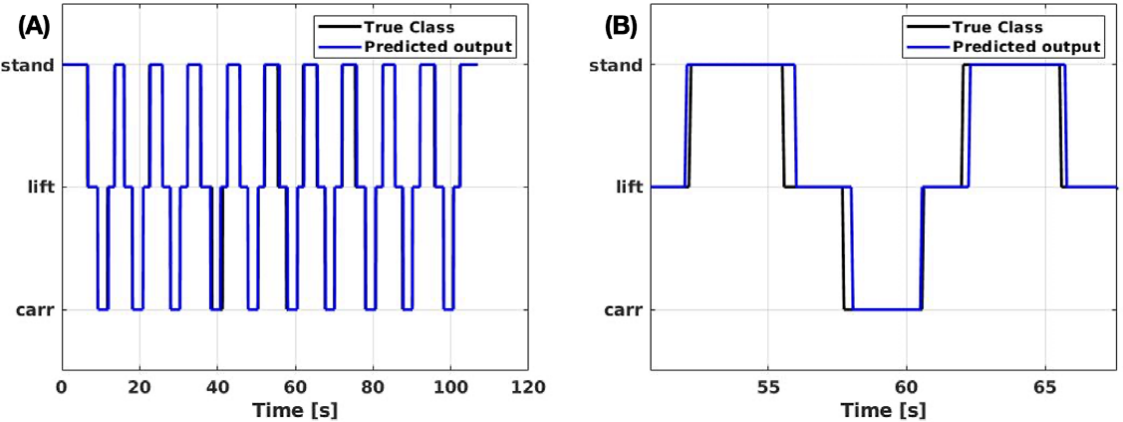
Plot of the online classification performance of the classifier, for subject 3. The black line represents the ground truth labeling, whereas the predictions are marked in blue. (a) Total overview of XoHar condition and (b) transition details.

**Figure 8.**
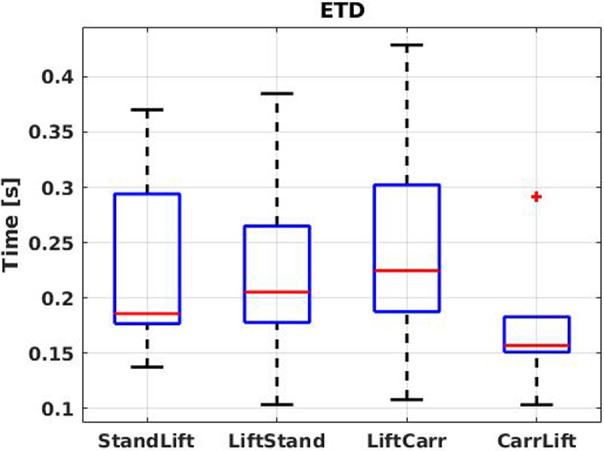
Boxplot representation of the classifier ETD for all the subjects.

A balanced classification problem is one where each class has, approximately, the same number of occurrences of the others (Wei and Dunbrack, 2013). Therefore, considering both the task description and the true class labeling (sum of each row), it emerges that the classification problem is well balanced. As a consequence, it is important to properly recognize each task. The accuracy value, computed analyzing the CM diagonal terms, is 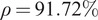 . Apart from lifting precision ( 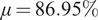 ), precision and recall values are always greater than 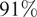 . More in details, when the subjects were performing lifting, the classifier almost misclassified the same amounts of samples ( 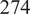 , corresponding to 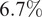 of the total lifting labels and 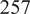 , corresponding to the 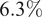 ), interpreting them as standing or carrying, respectively. This is a problem because, due to wrong activity recognition, the exoskeleton did not deliver the proper torque assistance. However, taking into account the lifting and lowering efficacy results (see Table 2) this did not seem to affect the exoskeleton performance (only 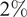 difference between XoLift and XoHar). A possible explanation of this result is that the misclassification occurred only at the transition points, where errors have less negative impact. Indeed, another element that should be taken into consideration is that the classification errors are not due to completely wrong recognitions (e.g., in standard problems wrongly recognizing a dog picture as a cat), but rather classification delays. As an example, the classification online performance for subject 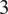 is reported in Figure 7. For this particular subject, it appears like the predicted lift 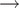 stand transition (LiftStand) sometimes anticipates the true transition ( 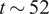 s) and sometimes is delayed ( 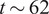 s). Instead, the stand 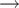 lift (StandLift) and the carr 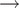 lift (CarrLift) transitions are delayed, confirming the hypothesis made above on the unassisted initial movements (see 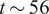 , 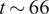 , and 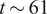 s). For more details on the ETD of the classifier, Figure 8 reports the boxplot representation of the average ETDs for all the subjects, calculated without discriminating between anticipations or delays. It is worth pointing out that the CarrLift transition is the one with less variability among the subjects.

#### Subjective questionnaire

**Figure 9.**
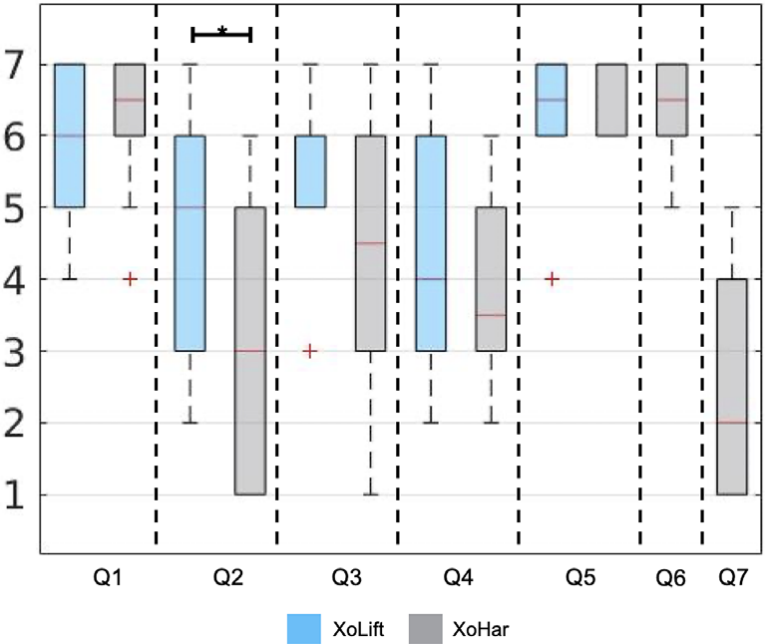
Questionnaire results. The questions are reported below for an easier interpretation. Horizontal segments link conditions where the difference was statistically significant (* < 5%). For Q1–Q6 the possible answers were 1 (Totally Disagree)—7 (Totally Agree). For Q7, instead the possible answers were 1 (Never)—7 (Always). Q6 and Q7 were answered only after the XoHar condition. Q1: Overall, do you feel less fatigued with respect to the noExo (control) condition? Q2: Did you feel that the exoskeleton action was constraining/hindering the natural leg movements, with respect to the noExo (control condition)? Q3: Did you feel that the exoskeleton action was providing assistance during the carrying activity? Q4: Did you feel that the exoskeleton action was constraining/hindering the natural trunk movements, with respect to the noExo (control condition)? Q5: Did you feel that the exoskeleton action was providing assistance during the lifting activity? Q6: Is it useful that the exoskeleton automatically recognizes the activity being performed? Q7: How often would you like to manually set the activity being performed?

The subjective questionnaire analysis, supports the results discussed referring to the kinematic and muscle activity data. The results are reported in Figure 9. In particular, the high scores obtained in Q1 for both exoskeleton conditions, can indicate that wearing XoTrunk the subjects fatigued less with respect to the noExo condition. In the XoLift case, the most frequent answer was *Slightly Agree* (score 5), indicating a mild perceived assistance. For the XoHar case, instead, the most frequent answer was *Totally Agree* (score 7), underlining a stronger perceived assistance. The difference between these conditions was not statistically significant, though.

Q2 investigated whether the test subjects felt that the exoskeleton assistance was hindering the leg movements. Here, as expected, the worst perceptions were associated with XoLift, whereas XoHar is the condition were the users reported less hindrance. The differences between the two conditions reached statistical significance, underlying the advantage of a versatile exoskeleton.

Q3 was related to the assistance that users experienced during the carrying activity. Again, in line with the expectations, XoLift can be associated with the condition delivering the highest assistance, and, interestingly, some subjects perceived assistance also in XoHar. The differences were not statistically significant.

Q4 was on trunk hindrance and there are no significant differences among the conditions. This is not a surprise as the mid-level control strategy was the same. Here, it is interesting to note how several subjects felt that their trunk movements were being constrained by the exoskeleton, supporting the kinematic analysis conducted in section “Dynamic Fit.”

Q5 was about the assistance perceived during lifting and the boxplots clearly indicate that there were no differences—related to the control strategy—in the subjective perception.

Q6 and Q7 did not compare different conditions but tested whether the automatic activity recognition is a valuable feature to the users. It appears that none of the test subjects considered HAR to be superfluous and that manually operating a switch is an operation that, if possible, should be avoided.

This questionnaire was done after replicating a real working condition scenario and, thus, the findings appear to be encouraging toward an adoption of versatile exoskeletons in the workplace. However, it should not be forgotten that in this study the subjects did not wear the exoskeleton for long time intervals and, so, generalizing these results to the actual industrial scenario is nontrivial. Finally, it should be mentioned that we are currently working on a study to validate the questionnaire comparing the results with those obtained via SUS, Borg-CR10 and Nasa-TLX scales (Hart and Staveland, 1988; Borg, 1990; Jordan et al., 1996).

## Field Testing

The XoTrunk exoskeleton analyzed in this study, was also used during field testing. The testing involved three companies were MMH activities are performed on a daily basis. In these sites, several tasks where the exoskeleton assistance would be beneficial were tested. This section describes some of the preliminary feedbacks related to the activity, simulated and presented above (see section “Task Description”). The field trials involved four workers for a duration of 9 hours, distributed in 4 days of testing (roughly 2 hours per worker). During the trials, we had the possibility to interact with the users and collect, by means of unstructured interviews, their feedbacks on the exoskeleton usage. We were particularly interested in understanding if the workers perceived that, while wearing the exoskeleton, they could work in a natural way and receive appropriate assistance. At first, we started the field testing with the XoLift condition. After about 20 min of usage all the users got used to the exoskeleton assistance and reported that they felt the exoskeleton was following their movements in an appropriate manner. However, as the users got used to the device, they became more aware of nonidealities in the assistive strategy. In particular, in the XoLift condition, the users reported that the exoskeleton was *“a bit too sensitive.”* This was said referring to the fact that, due to the EMG component in the control, the exoskeleton provided assistive torques also in undesired situations, like when typing on a keyboard or wrapping products. Such negative impressions were drastically reduced when switching to the versatile control (XoHar). In this situation, the workers really appreciated that the exoskeleton was providing assistive torques only when needed. A very nice feedback was from a user that said: *“now the exoskeleton feels like a part of my body.”* The fact that the exoskeleton was capable of well adapting to the user movements, also suggests that the classifier performance were adequate for the working scenario. More in particular, the classifier identified lifting for 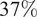 of the time, carrying for only 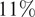 of the time and standing for the remaining 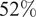 of the time. In sections “Task Description” and “Classifier performance,” it was presented how the task was well balanced between lifting/lowering, carrying and standing. This was not the case for the field testing, hence, it might be possible to conclude that the classifier was not properly generalizing to *out-of-the-laboratory* activities. However, it should also be mentioned that in the actual scenario, workers did not always have to travel 3.5 m and, sometimes, the carried distances were lower, partially explaining why carrying was not as frequent as the other activities. Therefore, these considerations, together with the subjective perception of the workers, can be used to hypothesize that the classifier performance were adequate. At the end of the trials, some of the collected feedbacks included appreciation for the assistance provided by the exoskeleton: *“for workers like us, this exoskeleton is a true cure-all”* or *“if available, I’d like to use this device every day.”* These feedbacks are really encouraging as they suggest that combining assistance and proper control strategies, the workers are willing to adopt exoskeletons. However, the workers were also concerned about the weight of the exoskeleton and the thermal conditioning, especially when removing the device. Finally, it should also be noted that the reported results can represent only a preliminary outcome. Indeed it should be stressed that the workers pool was very low (four subjects) and no worker used the exoskeleton for more than 2.5 hr.

## Conclusion

The aim of this work was to understand the advantages that a versatile exoskeleton has with respect to a non-versatile one. This was done, at first, in the laboratory and then in an operational environment. In particular, in the laboratory we simulated a MMH activity, as seen in a food manufacturing plant. To do so, we asked 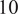 subjects (five males and five females) to perform successive repetitions of lifting, carrying and lowering of a 11.4 kg load, under three conditions: no exoskeleton, exoskeleton with non-versatile control strategy and exoskeleton with versatile control. The study was carried out with XoTrunk, the back-support exoskeleton developed at the Istituto Italiano di Tecnologia (IIT). The analyzed metrics focused not only on the efficacy of the device but also on its dynamic fit, that is, how user and device interact one with the other. Efficacy was evaluated focusing on the reduction of back muscle activation during the execution of lifting, lowering, and carrying. The results showed that XoTrunk offers remarkable reductions of muscle activation not only in lifting/lowering (up to 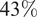 ) but also in carrying (up to 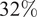 ). The versatile condition was associated with lower muscle activation reductions in carrying as it was controlled in order to provide null interaction torques between the user and the device itself. Apparently, this result seems to penalize the versatile exoskeleton condition. However, once dynamic fit is considered the situation changes. Indeed, thanks to the exoskeleton versatility, the users perceived less hindrance, assessed via subjective questionnaires. Also, the kinematic analysis of the trunk and hips joints complements these findings. Efficacy and dynamic fit results should be used to guide the design of a carrying assistive strategy that, exploiting versatility, should aim at consistent muscle activation reductions (XoTrunk potential is around 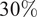 ) with low or null movement impairments. Of course, in order to develop carrying strategies it is necessary to properly recognize when this activity is taking place. The classifier used in this study proved to be reliable in the laboratory (online classification accuracy 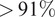 ). Moreover, even though during the field testing it was not possible to quantify the algorithm performance, no worker complained about possible activity misclassifications, suggesting it generalized well to out-of-the-lab scenarios. During the 9 hours of field testing, it was possible to collect feedbacks and impressions from the four workers that were involved. These workers expressed a general preference for the versatile exoskeleton condition. This supports the hypothesis that a versatile exoskeleton could help promote the adoption of these devices and, consequently, mitigate the risk of injuries associated with MSDs.

## Data Availability

The data that support the findings of this study are available from the corresponding author, T. Poliero, upon reasonable request.

## Footnotes

1

https://dati.inail.it/opendata/

2

https://www.cdc.gov/niosh/engcontrols/ecd/detail25.html

3

https://advr.iit.it/index.php/projects/inail-scc

4
Data reported in the mean  SD format.

5

http://www.seniam.org/

6
For more information, the reader is invited to refer to Section 23.4 of the Xsens MVN User Manual, Revision Z, 01 04 2021.
