# Versatile and non-versatile occupational back-support exoskeletons: A comparison in laboratory and field studies – ADDENDUM

**DOI:** 10.1017/wtc.2023.27

**Published:** 2024-02-12

**Authors:** Tommaso Poliero, Matteo Sposito, Stefano Toxiri, Christian Di Natali, Matteo Iurato, Vittorio Sanguineti, Darwin G. Caldwell, Jesús Ortiz

The authors regret the omission of a disclaimer that should have been included in the original funding statement. The sentence is provided below:

‘The content of this work reflects only the author’s view, and that the JU is not responsible for any use that may be made of the information it contains.’

The original article has been updated.

